# Interaction of functional *NPC1 *gene Polymorphism with smoking on coronary heart disease

**DOI:** 10.1186/1471-2350-11-149

**Published:** 2010-10-18

**Authors:** Weiwei Ma, Jing Xu, Qianqian Wang, Ying Xin, Lin Zhang, Xinxin Zheng, Hu Wang, Kai Sun, Rutai Hui, Xiaohong Huang

**Affiliations:** 1 Department of Cardiology, and Sino-German Laboratory for Molecular Medicine, Key Laboratory for Clinical Cardiovascular Genetics, Ministry of Education, FuWai Hospital and Cardiovascular Institute, Chinese Academy of Medical Sciences and Peking Union Medical College, 167 Beilishilu, 100037 Beijing, China; 2 Fu Wai Hospital and Cardiovascular Institute, Chinese Academy of Medical Sciences and Peking Union Medical College,167 Beilishilu, 100037 Beijing, China

## Abstract

**Background:**

The protein of Niemann-pick type C1 gene (*NPC1*) is known to facilitate the egress of cholesterol and other lipids from late endosomes and lysosomes to other cellular compartments. This study aims to investigate whether the genetic variation in *NPC1 *is associated with risk of coronary heart disease (CHD) and to detect whether *NPC1 *might interact with smoking on the risk of CHD.

**Methods:**

We performed a case-control study, including 873 patients with coronary heart disease (CHD) and 864 subjects without CHD as control. Polymorphisms of *NPC1 *gene were genotyped by polymerase chain reaction (PCR) -restriction fragment length polymorphism (RFLP).

**Results:**

A tag-SNP rs1805081 (+644A > G) in *NPC1 *was identified. The G allele of the +644 locus showed reduced risk of CHD than wild-type genotype in Chinese population (recessive model GG vs. AG+AA: odds ratio [OR] 0.647, 95% CI 0.428 to 0.980, *P *= 0.039; additive model GG vs. AG vs. AA: OR 0.847, 95% CI 0.718 to 0.998, *P *= 0.0471). Moreover in smokers, the G-allele carriers had reduced risk of CHD compared with A-allele carries (OR 0.552, 95% CI 0.311 to 0.979, *P *= 0.0421).

**Conclusions:**

The results of the present study suggest that *NPC1 *variants seem to be contributors to coronary heart disease occurrence in Chinese population. Moreover, in smokers, *NPC1 *variants seem to confer protection to coronary heart disease.

## Background

Coronary heart disease is one of the leading causes of morbidity and mortality in China. It is still a big challenge for medical society to elucidate the pathogenesis and to identify the subjects at risk. Coronary heart disease develops dependent of genetic predisposition and the accumulation of risk factors that lead to a wide array of molecular and cellular abnormalities within the vessel wall. It is well established that the formation of foam cell or atherosclerotic plaque is the hallmark event leading to coronary heart disease, Foam cells in atherosclerotic lesions derive from macrophages and vascular smooth muscle cells (VSMCs)[1]. Free cholesterol (FC) accumulation in lesional foam cells has been well documented in advanced atherosclerosis, and studies with cultured macrophages have shown that acceleration of FC accumulation in lesional macrophages in mice leads to increased lesional macrophage apoptosis[2-4]. Delivery of free cholesterol from the endocytic pathway to the plasma membrane and endoplasmatic reticulum (ER) requires the coordinated actions of the late endosomal Niemann-pick type C1 protein (NPC1). NPC1 protein inactivation results in lipid accumulation in late endosomes and lysosomes, leading to a defect of ATP binding cassette protein A1 (Abca1)-mediated lipid efflux to apolipoprotein A-I (apoA-I) in macrophages and fibroblasts[5].

NPC1 protein is a membrane protein localized in the late endosomes and regulates intracellular cholesterol trafficking [6]. Loss of *NPC1 *function leads to defective suppression of sterol regulatory element binding protein (SREBP)-dependent gene expression and failure to appropriately activate liver X receptor-mediated pathways, ultimately resulting in intracellular cholesterol accumulation in macrophages and fibroblasts[7,8]. Niemann-Pick type C disease is an autosomal, recessive disorder characterized by intracellular accumulations of cholesterol and other lipids throughout the body. Affected individuals exhibit progressive neurodegeneration, leading to death in childhood, adolescence, or early adulthood. The underlying gene, *NPC1 *responsible for 95% of Niemann-Pick type C disease, encodes a multispanning membrane protein containing a sterol-sensing domain that is required for the egress of cholesterol from late endosomes [9].

Cigarette smoking is a recognized risk factor for cardiovascular diseases and has been implicated in the pathogenesis of atherosclerosis[10]. Aryl hydrocarbons (AHs) such as 2,3,7,8-tetrachlorodibenzop-dioxin (TCDD) and polycyclic AHs (PAHs), which are especially found in cigarette smoking, had been recently demonstrated to promote the transformation of macrophages to foam cells in the atherosclerosis process [11]. Benzo(a)pyrene(BP)[12], a prototypical PAH, has thus been shown to accelerate atherosclerotic plaque development in apolipoprotein-E knock-out mice, via promoting a local inflammatory response, and inducing DNA damage in human blood vessels. In TCDD- and BP-exposed macrophages the *NPC1 *expression decreased at both mRNA and protein levels.

*NPC1*-deficient cells exhibit a defect in the intracellular trafficking of low-density lipoprotein-derived cholesterol, leading to cholesterol accumulation in late endosomes and lysosomes and defects in endoplasmic reticulum-initiated cholesterol regulatory events[13]. Some scientists [14,15] have reported that mice carrying a naturally occurring null mutation of *NPC1 *have a defect in apoA-1-mediated cholesterol efflux from macrophages. This underlines the relationships between *NPC1*, cholesterol homeostasis, and lipid accumulation and cigareete smoking. The aim of this study was to look at the association and linkage between the *NPC1 *gene polymorphism and coronary heart disease in a Chinese population. In addition, the relationship of the *NPC1 *gene polymorphism with cigarette smoking was assessed.

## Methods

### Study sample

The study was approved by the institutional review board of cardiovascular institute, and the participating hospitals. All subjects who participated in the study provided their written informed consent and were self-reported as Han nationality. The subjects of the study consisted of 873 CHD patients and consisted of 864 control subjects without CHD. The 873 case subjects were recruited from FuWai Hospital from December 2004 to December 2006. Inclusion criteria was >70% narrowing of the lumina of at least 1 major coronary artery according to their coronary angiography (CAG) results. A detailed history of angina or MI was obtained. Patients with MI were judged by typical ECG change (Minnesota Code 1.1 or 1.2 in ECG) and by changes in serum enzymes (troponin T, troponin I, creatine kinase-MB, aspartate aminotransferase, and glutamic pyruvic transaminase)[16].

The 864 control subjects were recruited from 24 communities in China, whose major coronary arteries had no more than 20% stenosis and who did not have any vascular disease.

### Biological variable determination and clinical data collection

Blood samples were collected after a 12-hour overnight fast before cardiovascular procedures. In subjects with an acute event, the drawing of blood was delayed for at least 6 weeks. The plasma and cell buffet coat were kept at -70°C. Genomic DNA was extracted, and biological variables were determined within 3 months. A complete clinical history was obtained from all subjects. In addition to family history of hypertension[17], MI, and diabetes mellitus (DM), the following vascular risk factors were also recorded: history of vascular disease, cigarette smoking[18], body mass index (BMI), systolic blood pressure (SBP), diastolic blood pressure (DBP), blood glucose, high density lipoprotein cholesterol (HDL-C), low density lipoprotein cholesterol (LDL-C), total plasma cholesterol (TC), and triglycerides (TG). Hypertension was defined as a mean of 3 independent measures of blood pressure ≥140/90 mm Hg or the use of antihypertensive drugs. DM was diagnosed when the subject had a fasting glucose level ≥7.0 mmol/L, ≥11.1 mmol/L at 2 hours after oral glucose challenge, or both[19].

### SNP selection

SNPs in *NPC1 *gene were retrieved from HapMap database for CHB (Chinese Han in Beijing) sample (release No. 24/phaseII Nov08, on NCBI B36 assembly). We searched across a 47-kb region spanning *NPC1 *gene from 1 kb upstream of the 5'-flanking region to 0.5 kb downstream of the 3'-flanking region. The percent coverage of HapMap variants is subjected to a minor allele frequency (MAF) threshold of 0.05 and an r^2 ^> 0.8 threshold with pair-wise tagging, using Haploview software (3.31 version). After excluded those SNPs in introns, we identified one important SNP rs1805081 (+644A > G) with a MAF of 0.374, which causes a missense mutation from His215 to Arg in NPC1 protein.

### DNA preparation, PCR, and PCR-based allele genotyping

Genomic DNA was isolated from peripheral blood leukocytes by the FlexiGene DNA Kit (Qiagen, Hilden, Germany). Genotyping was carried out by PCR-RFLP analysis. Primers were designed with Oligo 6 software as following: 5'-GGGTTGCCTTGGTATGTG-3' and 5'-GATCGTCCAGGGAGCAG-3'. The reaction mixture was subjected to denaturation at 95°C for 5 min, followed by 34 cycles at 94°C for 20 sec, 60°C for 30 sec, 72°C for 35 sec, then by a final extension at 72°C for 5 min. The resultant polymerase chain reaction (PCR) products were digested with NcoI (New England Biolabs, Beverly, Mass), which yielded 2 DNA fragments of 269 and 143 bp for the A allele on 3.5% agarose gel and only one 412 bp band for the G allele. Reproducibility of genotyping was confirmed by bidirectional sequencing in 50 randomly selected samples, and the reproducibility was 100%.

### Statistical Analysis

Quantitative variables, including age, body mass index, SBP and DBP, glucose, HDL-C, LDL-C, and TC, were compared with the 1-way ANOVA test. A χ^2 ^test was used to test for qualitative variables, genotype/allele frequencies, and Hardy-Weinberg equilibrium of the polymorphisms. ORs and 95% CIs were computed with the use of binary logistic regression analyses. Adjusted ORs were stratified by age, sex, body mass index, hypertension history, cigarette smoking, and Diabetes mellitus history. Gene-envirionment interaction were calculated by binary logistic regression analyses and software for calculating measures of biological interaction[20]. A 2-tailed probability value of ≤0.05 was considered significant. Analyses were performed with SAS9.1 for Windows (Microsoft Corp, Redmond, Wash).

## Results

### G allele of tag SNP rs1805081 was associated with risk of CHD

Clinical characteristics of the subjects in the present study are shown in Table 1. In this group, significantly higher ratio of men, higher levels of TG, lower level of HDL-C, higher cigarette smoking rate and higher incidence of DM were found in cases than in controls (all *P *< 0.01). The distribution of the genotypes of tag SNP rs1805081 is shown in Tables 2. All genotypes conformed to Hardy-Weinberg equilibrium expectation in this group. The genotype frequency of the rs1805081 from the HapMap data was similar to ours. We analyzed the association of rs1805081 with CHD using both a dominant model and an additive model. The genotype frequencies of G allele homozygote and heterozygote were significantly lower in patients with CHD than in control subjects, and the protective allelic frequencies were also lower in CHD patients than in control subjects (Table 2). The association remained after adjustment for by age, sex, body mass index, hypertension history, cigarette smoking, Diabetes mellitus history with binary logistic regression analysis, supporting that G allele of tag SNP *NPC1 *was a protective factor for CHD. As shown in Table 3, in this population, the odds ratio [OR] was 0.647 (95% confidence interval [CI] 0.428 to 0.980, *P *= 0.039) under a recessive model (GG vs. AA+AG) and 0.847 (95% confidence interval [CI] 0.718 to 0.998, *P *= 0.047) under an additive model (GG vs. AG vs. AA).

**Table 1:** Clinical Characteristics of the Population

Characteristics	CHD	Controls
N	873	864
age, year**	51 (8.14)	53 (8.39)
Men,n (%)*	734 (84.0)	612 (70.8)
Body mass index, kg/m^2^	25.57(3.20)	24.95(9.37)
HDL-C, mmol/L**	1.18 (0.37)	1.35 (0.35)
LDL-C, mmol/L**	2.37 (0.78)	2.75 (0.85)
TC, mmol/L**	4.47 (1.05)	5.03 (1.10)
TG, mmol/L**	1.34 (1.26)	1.12 (1.43)
Cigarette smoking, n (%)**	582 (66.67)	351 (40.91)
Hypertension history, n (%)	431 (49.39)	412 (47.74)
Diabetes mellitus history, n (%)**	183 (20.96)	67 (7.75)

Clinical characteristics of age, Body mass index, LDL-C, HDL-C, TC and TG values are given as mean (SD); and other values as number of individuals.

*P < 0.05, **P < 0.01 vs. control

**Table 2:** The distribution of the genotypes of rs1805081 in *NPC1*

	Genotype,n(%)			Allele, n(%)	
	
Groups	AA	AG	GG	A	G
Controls(n = 864)	479(55.44)	316(36.57)	69(7.99)	1274(73.73)	454(26.27)
Cases(n = 873)	512(58.65)	314(35.79)	47(5.38)	1338(76.63)	408(23.37)

**Table 3:** Association of rs1805081 in *NPC1 *Gene with CHD in the Population

Models	Crude ORs(95% CI)	Adjusted ORs(95% CI)	Adjusted *P *Value
GG vs. AG+AA	0.656(0.447 to 0.962)	0.647(0.428 to 0.980)	0.0399
GG vs. AG vs. AA	0.859(0.738 to 1.001)	0.847(0.718 to 0.998)	0.0471
GG+AG vs. AA	0.877(0.725 to 1.061)	0.860(0.701 to 1.055)	0.1489

ORs and 95% CIs were computed with the use of binary logistic regression analyses. Adjusted ORs were stratified by age, sex, body mass index, hypertension history, cigarette smoking, and Diabetes mellitus history.

### *NPC1 *ploymorphism interact with smoking on coronary atherosclerosis

The odds ratio of smoking alone on CHD risk was 2.42 (95% CI 1.959 to 2.988, *P *< 0.0001). To determine whether smoking influenced the effects of gene load on coronary heart disease. We analyzed the association of +644A/G with CHD by two group, nonsmoking and smoking. Among smokers, in recessive model (GG vs. AA+AG) and additive model (GG vs. AG vs. AA) the genotype frequencies of G allele homozygote and heterozygote were lower in CHD patients than in control subjects (table 4). The ORs were adjusted by age, sex, body mass index, hypertension history, Diabetes mellitus history.

**Table 4:** Association of rs1805081 in *NPC1 *Gene with CHD classified by smoking

		non-smoking		smoking	
		
Models		ORs(95% CI)	*P *Value	ORs(95% CI)	*P *Value
GG vs. AG+AA	Crude	0.773(0.441 to 1.357)	0.3703	0.606(0.351 to 1.047)	0.0727
	Adjusted	0.766(0.423 to 1.386)	0.3537	0.552(0.311 to 0.979)	0.0421
GG vs. AG vs. AA	Crude	0.954(0.759 to 1.198)	0.6839	0.810(0.652 to 1.006)	0.0571
	Adjusted	0.909(0.714 to 1.158)	0.441	0.778(0.620 to 0.976)	0.0301
GG+AG vs. AA	Crude	0.994(0.744 to 1.328)	0.9672	0.817(0.625 to 1.068)	0.1391
	Adjusted	0.846(0.622 to 1.151)	0.2868	0.804(0.607 to 1.065)	0.1277

ORs and 95% CIs were computed with the use of binary logistic regression analyses. Adjusted ORs were stratified by age, sex, body mass index, hypertension history, cigarette smoking, and Diabetes mellitus history.

The interaction effect between +644A/G and cigarette smoking were calculated in two most widely used modes including multiplicative interaction mode and additive interaction mode. In multiplicative interaction mode, there was a significant interaction between *NPC1 *genotype and cigarette smoking on risk for CHD (*P *for interaction = 0.0001). Using four-by-two table to estimate additive interaction between +644A/G and cigarette smoking, we found no interaction between *NPC1 *genotype and cigarette smoking on risk for CHD (Table 5).

**Table 5:** The additive interaction of rs1805081 in *NPC1 *Gene with CHD in the Population

	Modle	
	
measure(95% CI)	recessive	additive
RERI	-0.240 (-0.913 to 0.433)	-0.802 (-1.784 to 0.180)
AP	-0.128 (-0.493 to 0.237)	-0.626 (-1.662 to 0.410)
SI	0.786 (0.417 to 1.481)	0.260 (0.018 to 3.687)

RERI(relative excess risk due to interaction), AP (attributable proportion due to interaction) and SI (synergy indes) with CI (confidence intervals). Lack of interaction is reflected by RERI = AP = 0 and SI = 1.

## Discussion

The present study provides evidence that +644A > G, a common variant of *NPC1*, was associated with a decreased risk of CHD patients in recessive and additive model. Moreover, in smokers, carriers of *NPC1 *GG had a decrease risk of coronary heart disease as compared with those carrying AA and AG. Multivariate adjustments support that the effects are independent of common traditional cardiovascular risk factors, including age, sex, body mass index, hypertension history, Diabetes mellitus history. Hypercholesterolemia is traditional cardiovascular risk factor of CHD. However, in this study the CHD patients almost took cholesterol-lowering drugs to control cholesterol levels. Therefore compared with control, LDL-C and TC were significant lower and HDL-C was significant higher in CHD patients (*P *< 0.01). Because of cholesterol-lowering drugs influences, in the logistic regression model the results did not adjusted by the classical risk factor LDL-C, HDL-C, TG and TC.

A connection between the *NPC1 *and atherosclerosis has been established in several studies. Deletion of *NPC1 *in BM-derived cells accelerated atherosclerosis by impairing cholesterol efflux and promoting cellular oxidative stress. Jessie R. Zhang et al. disrupted *NPC1 *expression in macrophages of *Ldlr*^*-/- *^mice and demonstrated that absence of *NPC1 *expression was proatherogenic *in vivo*[21]. Macrophages from mice with *NPC1 *heterozygous mutation have a selective defect in cholesterol trafficking to the ER and are protected from cholesterol-induced apoptosis[22]. Our study indicated that the variant allele G at position +644 in *NPC1 *is associated with decreased risk of CHD. The *NPC1 *cholesterol trafficking pathway could have an impact on the progress of atherosclerosis by above mechanisms. Furthormore, rs1805081 has been found to be associated with obesity[23] and Alzheimer's[24] while studies in mice suggest that the G allele is protective here and might be associated with NPC1 activity. Moreover, the *NPC1 *cholesterol trafficking pathway regulates liver X receptor-dependent cholesterol efflux in vivo, alleviating cholesterol-induced oxidative stress and serving an atheroprotective role. The *NPC1*^*-/- *^chimeric mice exhibited increased atherosclerotic lesion formation despite lower cholesterol and TG levels in the VLDL and LDL fractions. *NPC1 *heterozygosity was associated with resistance to lesional necrosis and lesional macrophage apoptosis. More recently, Welch et al. [25] reported that introduction of the *NPC1*^*-/- *^mutation into the *Apoe*^*-/- *^background predisposed to increased lesion formation and atherothrombosis.

Smoking is known to raise serum lipid levels, and is considered as one of the major risk factors for the development of atherosclerosis disease. In the present study, we showed that within non-smokers, the coronary heart disease risk did not differ between *NPC1 *genotypes. In smokers, however, both in recessive model (GG vs. AA+AG) and additive model (GG vs. AG vs. AA) *NPC1 *G carriers showed decreased coronary heart disease risk compared with A carriers. Furthermore in multiplicative interaction mode, there was a significant interaction between *NPC1 *+644A > G and cigarette smoking on risk for CHD. However, the biological basis of *NPC1*-smoking interaction on lipid concentration and CHD risk is still unclear. Smoking might lower *NPC1 *transcription, by somehow disrupting the binding of transcription factors, therefore, leading to intracellular cholesterol accumulation. Normand Podechard[26] recently found that human macrophages exposed to environmental aryl hydrocarbons and Aryl hydrocarbons (AHs) was found to decrease expression of *NPC1 *at both mRNA and protein levels. Moreover 2,3,7,8-tetrachlorodibenzop-dioxin (TCDD) and polycyclic AHs (PAHs), which are especially found in cigarette smoking, had been recently demonstrated to promote the transformation of macrophages to foam cells in the atherosclerosis process[11].

We also examined whether the disease-associated alleles were related to specific vascular risk factors, including age, sex, body mass index, hypertension history, Diabetes mellitus history. After adjusting for those conventional cardiovascular risk factors, the association remained significant, suggesting that the contribution of this variant to the risk of CHD is independent of conventional cardiovascular protective factors.

The strength of the present study is the large sample size, and community-based sample. However, there are some limitations in the present study, including that there isn't the second case-control study to replicate the first study results. Moreover, a longitudinal follow-up study is required to elucidate whether there are protective effects of *NPC1 *variants on CHD in the future. In conclusion, a genetic variant of *NPC1 *could contribute to predict atherosclerotic and suggest therapeutic strategies to promote plaque stability. The results need to be replicated in other nationalities and confirmed with a prospective trial. The mechanisms need to be further explored.

## Conclusions

The results of the present study suggest that *NPC1 *variants seem to be contributors to coronary heart disease occurrence in Chinese population. Moreover, in smokers, *NPC1 *variants seem to confer protection to coronary heart disease.

## Competing interests

The authors declare that they have no competing interests.

## Authors' contributions

WM participated in study design, sample recruitment, DNA isolation, statistical analysis, interpretation of data and drafted the manuscript. JX participated in sample recruitment, DNA isolation and helped to draft the manuscript. QW and XZ participated in statistical analysis, in expectation of data. LZ participated in sample recruitment. YX participated in study design and DNA isolation. KS and HW participated in study design and manuscript revision. XH participated in study design, SNP selection, genotyping and manuscript revision.

All authors read and approved the final manuscript.

## Pre-publication history

The pre-publication history for this paper can be accessed here:

http://www.biomedcentral.com/1471-2350/11/149/prepub
